# Hornets Can Fly at Night without Obvious Adaptations of Eyes and Ocelli

**DOI:** 10.1371/journal.pone.0021892

**Published:** 2011-07-12

**Authors:** Almut Kelber, Fredrik Jonsson, Rita Wallén, Eric Warrant, Torill Kornfeldt, Emily Baird

**Affiliations:** Lund Vision Group, Department of Biology, Lund University, Lund, Sweden; Imperial College London, United Kingdom

## Abstract

Hornets, the largest social wasps, have a reputation of being facultatively nocturnal. Here we confirm flight activity of hornet workers in dim twilight. We studied the eyes and ocelli of European hornets (*Vespa crabro*) and common wasps (*Vespula vulgaris*) with the goal to find the optical and anatomical adaptations that enable them to fly in dim light. Adaptations described for obligately nocturnal hymenoptera such as the bees *Xylocopa tranquebarica* and *Megalopta genalis* and the wasp *Apoica pallens* include large ocelli and compound eyes with wide rhabdoms and large facet lenses. Interestingly, we did not find any such adaptations in hornet eyes or ocelli. On the contrary, their eyes are even less sensitive than those of the obligately diurnal common wasps. Therefore we conclude that hornets, like several facultatively nocturnal bee species such as *Apis mellifera adansonii*, *A. dorsata* and *X. tenuiscapa* are capable of seeing in dim light simply due to the large body and thus eye size. We propose that neural pooling strategies and behavioural adaptations precede anatomical adaptations in the eyes and ocelli when insects with apposition compound eyes turn to dim light activity.

## Introduction

Hornets, the largest of all social wasps, have not only fascinated humans by their size and painful sting, but also by the fact that they – in stark contrast to smaller sized vespids – can be observed flying at night. It is thus surprising that only two studies have been dedicated to their nocturnal life-style. Blackith [1] compared the morning start and evening end of activity of common wasps, *Vespula rufa*, *V. germanica* and *V. vulgaris* with those of *Vespa crabro*. He found that *V. crabro* fly earlier in the morning and later in the evening, at light levels that are about 100 times lower, than the other wasps (0.03 lumens/m^2^, as compared to 2.4 lumens/m^2^). Blackith [1] proposes that this allows them to fly in moonlit nights, with a full or three-quarter moon, when light intensities are around 0.1–0.2 lux. He concludes that light intensity is the limiting factor that keeps hornets from flying at even dimmer light levels. More recently, Spiewok and Schmolz [2] found that tethered hornet workers fly more slowly in lower light intensities (0.5 lux) than in bright light (850 lux).

Unlike in hornets, visual organs and visually guided behaviour in other nocturnal and crepuscular wasps, bees and ants have been studied in quite some detail (e.g. [3]–[9]). Kelber et al [5] conclude for crepuscular bees, just as Blackith [1] did for hornets, that light levels – or rather, the low sensitivity of their apposition compound eyes – limits their nocturnal activity.

The apposition compound eye of insects is built of several hundreds or thousands of visual units called ommatidia. Each ommatidium is optically isolated from its neighbours by light-absorbing screening pigment. The ommatidium consists of a corneal facet, a crystalline cone and a rhabdom, all surrounded by pigment cells. Light striking the ommatidial lens will only reach the underlying rhabdom and will not penetrate neighbouring rhabdoms. The small aperture of the facet lens limits photon capture and thus absolute sensitivity, making this eye-type less suitable for vision at night. However, several insect groups use apposition compound eyes in dim light, for instance crickets, grasshoppers, cockroaches and a limited number of hymenoptera [10]).

Detailed studies on the eyes and ocelli of the halictid bee *Megalopta genalis* [3], the paper wasp *Apoica pallens* [4], [11], nocturnal bull ants [6] and the nocturnal carpenter bee *Xylocopa tranquebarica* [7], [12] showed that enlarged ocelli and large compound eyes with relatively large facet lenses and unusually wide rhabdoms, were the common features for all of these nocturnal hymenoptera. These adaptations make their eyes about 30 times more sensitive to light when compared to the eyes of diurnal hymenoptera [3]. However, the light intensities at which some of these bees fly and navigate using visual cues and even discriminate colour, are millions of times dimmer than daylight [5], [12]. Additional neural mechanisms such as spatial and temporal pooling have therefore been assumed to take place [3], [13].

In the eyes of European honeybees, neural pooling mechanisms had been proposed to occur a while ago [14]. European honeybees are diurnal, but the African race, *A. m. adansonii*, can forage on nights when the moon is half-full or larger. The same is true for the Asian giant honeybee, *A. dorsata*, and for the Asian carpenter bee *X. tenuiscapa* (see 8, 12]. These species lack the optical and anatomical adaptations that have been observed in nocturnal bees.

What adaptations do we find in the eyes of hornets that are generally diurnal but can fly at night? And how regularly do hornets fly at night in places where no man-made illumination brightens the night? To answer these questions, we have studied the eyes and ocelli of the European hornet *Vespa crabro* and the common wasp *Vespula vulgaris*, and collected data on their nocturnal flight activity.

## Materials and Methods

### Animals and nest observations

For optical and anatomical studies hornets (*Vespa crabro*) and wasps (*Vespula vulgaris* ) were collected in southern Sweden during September 2006. Nest observations were made at three nests. Data were obtained from a video camera attached to the nest entrance of a nest at the Ammersee, in southern Germany during August and September 2007. This nest was very small, and observations were only used to confirm the other data sets. A second nest was observed during the evening of August 30^t^h, 2008, in southern Sweden (55° 48′ N, 14° 3′ E), with simultaneous light measurements using an International Light IL1700 radiometer. The nest entrance of the third nest, in southern Sweden (56° 40′ N, 14° 30′ E), was filmed with a video camera over four nights during August 2009 and during the bright hours of 10 days in August and September 2009. The number of hornets leaving or entering the nest during 5 minute slots were registered continuously for the videos taken between 30 minutes before sunset and 30 minutes after sunrise. For the daylight hours, only 5 minutes every hour were analysed.

### Histology

For light microscopy and transmission electron microscopy (TEM), eyes were dissected from the head and opened ventrally, to allow fixative to penetrate. The three ocelli were dissected together, attached to the underlying tissue. Eyes and ocelli were placed in fixative (2% paraformaldehyde, 2% glutaraldehyde, 2% sucrose 0.15 molar sodium cacodylate buffer) overnight, rinsed in buffer, postfixed in 1% OsO_4_ for one hour, dehydrated in an alcohol series and embedded in Epon.

For TEM, ultrathin transverse sections from the eye were stained with 2% uranylacetate and lead citrate and studied under a Jeol 1230 transmission electron microscope (Tokyo, Japan). For light microscopy, 3 µm thin longitudinal sections from both the eye and the ocelli were stained with Azur II-Methylenblue, and photos were taken using a Zeiss light microscope with an Olympus digital camera.

For scanning electron micrographs, entire heads of animals stored in 70% ethanol were dehydrated in an alcohol series, dried in air and gold-coated. Micrographs were taken under a Jeol LV 5600 SEM (Tokyo, Japan). The intertegular span, which is the thorax width measured between the wing bases, or tegulae of the animals, was used as a measure for body size [15]. Head width and head length are given for comparison with data in [6].

### Eye maps

Maps of interommatidial angles could only be obtained for *Vespa crabro* because no pseudopupil (see below) could be seen in *Vespula vulgaris*. The methods used to determine interommatidial angles are described in [3], [16]. Here, we give a short description.

The wasp was immobilized by gentle cooling, and fixed in position within a plastic pipette, the tip of which was cut open at the small end. The mouthparts were glued to the edge of the pipette and the neck joint was stabilised to prevent the animal from moving its head. The animal was then positioned with centre of the head at the centre of curvature of a Leitz goniometer, and the flat back edge of the eyes was aligned parallel to the stage. The three goniometer axes were lined up with the dorsal-ventral, anterior-posterior and left-right axes of the wasp's head. With the stage horizontal, the wasp's frontal visual field was oriented vertically upwards. The goniometer allowed us to tilt the wasp in defined steps of latitude and longitude, with the frontal direction defined as 0° latitude and 0° longitude, the latitude +90° corresponding to dorsal, the latitude −90° to ventral, and the longitude +90° to lateral. The goniometer was placed under a Canon MV650i camcorder mounted on a stand together with an inverted Hasselblad 80 mm objective.

The eyes of the animal were illuminated using a half-silvered mirror, angled at 45° just beneath the lens, and illuminated from the side by a light emitting diode. This way the eyes were illuminated and viewed along the same axis (‘‘orthodromic illumination’’), making a pseudopupil (the facets looking into the lens) visible. The goniometer was moved in steps of 10° and pictures were taken for all longitudinal and latitudinal positions. Due to obstacles in the experimental setup (e.g. the goniometer hitting the lamp holder), we could only study the frontal part of the eye extending to latitudes of 70° and longitudes of 80°. BaSO_4_ dust was sprinkled over the eye to provide landmarks that were used for determining the successive positions of the pseudopupil in the pictures.

From each of these photographs, and with the help of the landmarks, the coordinates of the facet at the centre of the pseudopupil were determined. Using established formulae to correct for latitude distortion in the projection, the average local interommatodial angle was calculated. The diameter of the facets was determined for each position. All these data were plotted on a sphere representing the three-dimensional space around the animal, and isolines were drawn by hand.

### Focal length

Focal length and back focal distance were determined using the hanging drop method first outlined by [17] and modified by [11]. A small piece of the facet cornea was carefully dissected from the fronto-ventral region, placed in a Petri dish with saline, cleaned with a paint-brush and placed with the external side outwards in small drop of saline on a cover slip. An o-ring was waxed onto a microscope glass slide and the upper surface of the o-ring was smeared with Petroleum jelly. The cover slip was then placed on the o-ring with the drop of saline carrying the lens facing downwards. This whole setup was inserted onto the stage of a Nikon light microscope with the condenser removed. A single facet lens was chosen for measurements, and objects of known size (striped patterns printed on translucent tracing paper) were placed over the lamp aperture in the foot of the microscope. The images formed inside the water drop by the facet were viewed through the microscope and photographed using a digital Canon camera. The focal length was calculated using the equation:
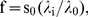
(1)where s_0_ is the distance between the object and the cornea (136 mm), λ_0_ is the spatial wavelength of the striped pattern (8 mm) and λ*_i_* is the spatial wavelength of the image (mm). Back focal distance was determined by first focusing on the debris on the back of the cornea and then moving the focus upwards until the focal plane was reached. The distance between the two points (in µm) was measured using a micrometer gauge attached to the microscope stage. The average of ten measurements was calculated and corrected for the refractive index of the used saline (1.33). The same procedure was used to measure focal lengths and back focal distances of the lateral and medial ocelli.

### F-number and optical sensitivity to white light

The F-number of the eyes is the ratio between focal length (*f*) and corneal facet diameter (aperture, *D*). Since both *f* and *D* are measurements of length, *F* is unitless.
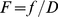
(2)


The optical sensitivity (*S*) is calculated as the amount of light that is absorbed by a photoreceptor when it views an extended source of white light [18], [19], and here we use the simplified formula developed by Warrant and Nilsson [20]:
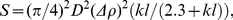
(3)where *D* is the corneal facet diameter (in µm), Δρ is the acceptance angle of the ommatidium (in radians), *l* is the rhabdom length (in µm) and *k* is the assumed absorption coefficient, taken to be 0.0067 µm^−1^ [21]. The sensitivity is thus given in units of µm^2^sr. The acceptance angle of the photoreceptor can be estimated as the ratio of *d* and *f* [22], [23]:
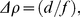
(4)where *d* is the distal rhabdom diameter, *f* is the focal length.

## Results

### Flight activity of Vespa crabro

**Figure 1:**
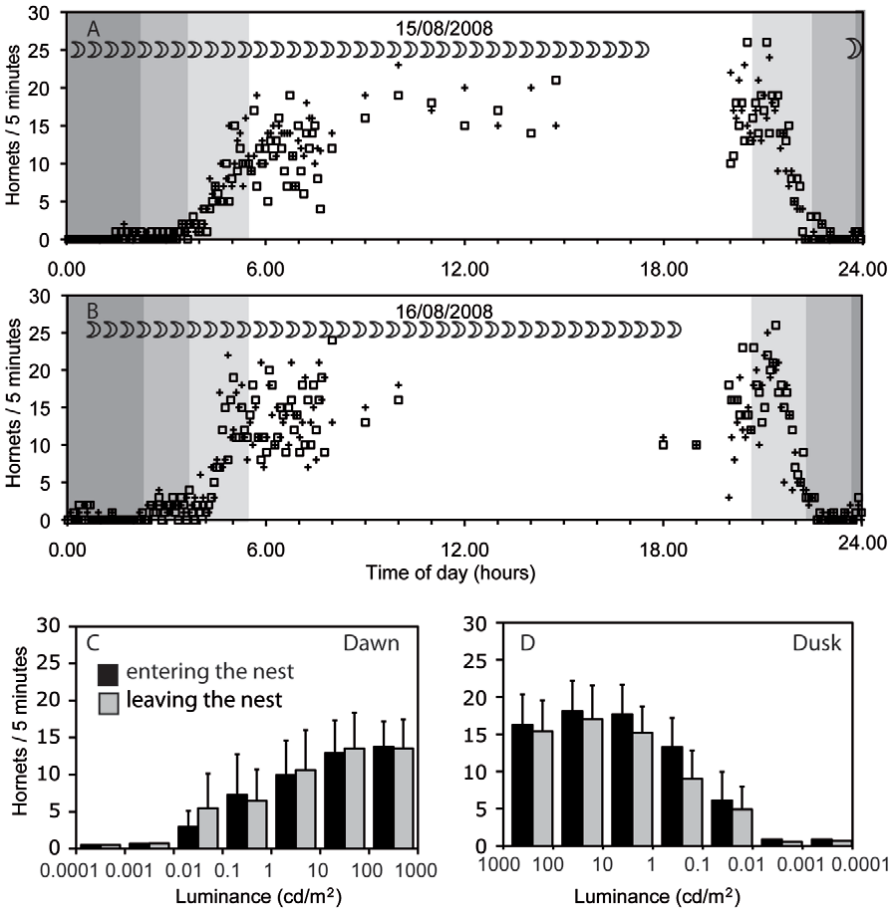
Flight activity of *Vespa crabro*. Data were recorded in Sweden, at 56°40′ North and 14°30′ East, during 4 nights between 14/8/2009 and 19/8/2009, and during 10 days in August and September 2009. **A**, **B** Examples of flight activity during two 24 hour periods. During the day, the number of bees entering (open squares) and leaving (crosses) the nest within five minutes was recorded every hour, for several hours per day. During the night, these numbers were recorded continuously. Dark grey shading indicates periods with no sunlight (sun more than 18° below the horizon), medium grey indicates time when the sun is between 12° and 18° below the horizon, and light grey shading indicates times when the sun is between 0° and 12° below the horizon. The moon was a waning crescent, and the moon symbols indicate when it was visible. **C, D** The average number (and standard deviation) of hornets entering (black bars) and leaving (light grey bars) the nest within five minutes, during dawn (**C**) and dusk (**D**) is given as a function of light intensities. Moonlight is in the range between 0.0001 and 0.01 Cd/m^2^ from a new moon to a full moon, depending on the elevation. Data from four nights and ten days.

Observations of flight activity were made during late summer at a nest in southern Sweden, where twilight lasts around three hours. The number of hornets flying from and to the nest was rather constant during the bright hours of day, from about one hour before sunrise until one hour after sunset, at light intensities of at least 1 cd/m^2^. On average, 15 hornets left or entered the nest during a five minute period, and activity increased somewhat in the early evening hours (Fig. 1A, B). Below this light intensity, and down to 0.001 cd/m^2^, activity decreased (Fig. 1C, D). Hornets rarely left and entered the nest at dimmer light levels.

### Eyes

**Figure 2:**
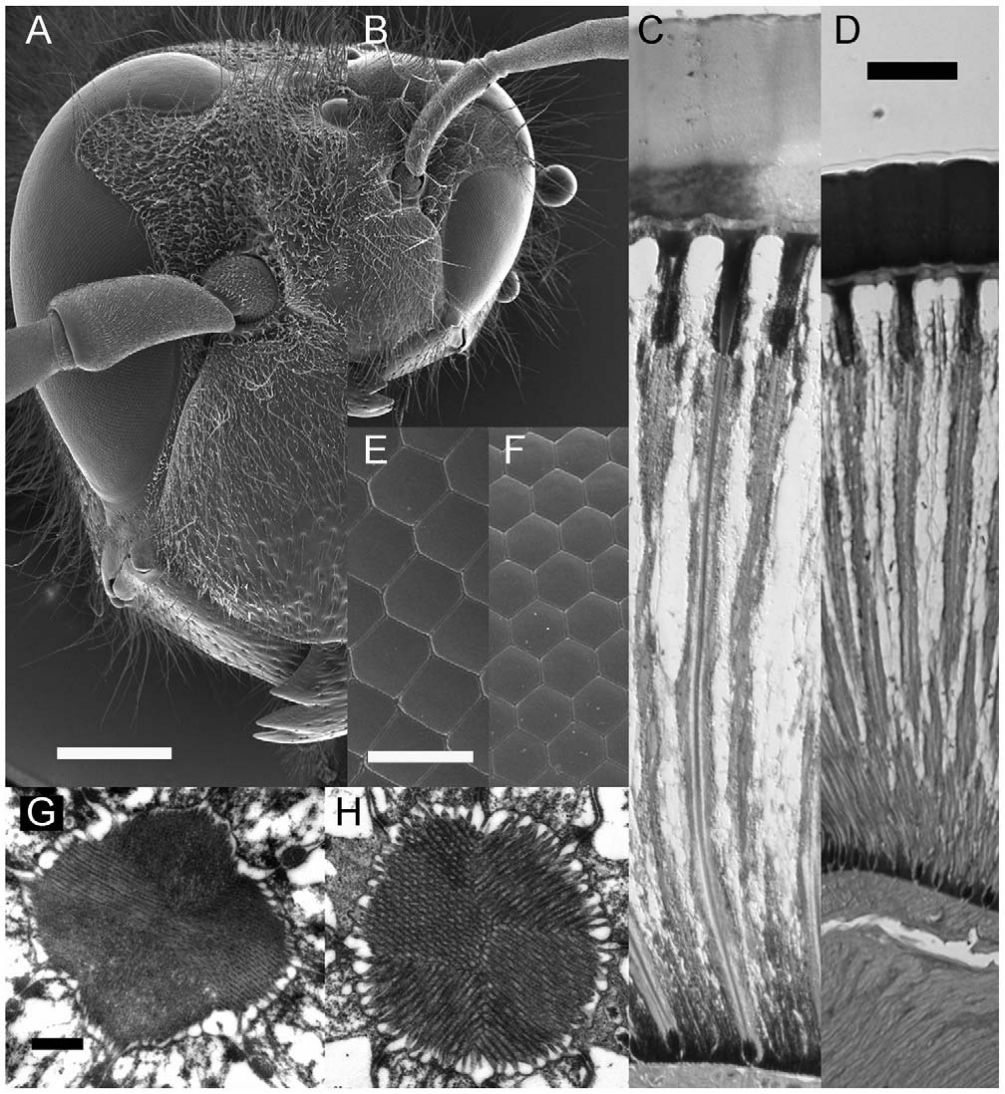
The eyes of *Vespa crabro* and *Vespula vulgaris* (left and right side, respectively, in each pair of figures). Each pair of figures has the same scale. **A**, **B** Frontal view of the head, note the cuticular indentation into the eye, scale bar 1 mm. **C**, **D** Longitudinal section through several ommatidia in the eye, showing a thick cornea, the crystalline cone, and the rhabdoms, scale bar 50 µm. **E**, **F** Facets of the compound eye, scale bar 50 µm. **G**, **H** Cross sections through the distal rhabdom, scale bar 0.5 µm.

*V. crabro* workers are much bigger (4.8 mm intertegular span) than *V. vulgaris* workers (2.5 mm intertegular span). These differences are also evident when comparing head and eye size (see Fig. 2A, B and Table 1). The head length for *V. crabro* is 6 mm, the head width is 5.6 mm and the eye length is 3.7 mm. The corresponding values for *V. vulgaris* are 3.2, 3.2 and 2.2 mm, respectively. Although much bigger in size, the eyes of *V. crabro* are smaller relative to body and head size than those of *V. vulgaris*.

**Table 1:** Body and eye measurements of four species of Vespidae.

	Symbol	Unit	Vespa crabro	Vespula vulgaris	Apoica pallens^1^	Polistes occidentalis^1^
Intertegular width		mm	4.8±0.1	2.5±0.1	2.7±0.1	
Head length		mm	6.0	3.2	3.1	3.7
Head width		mm	5.6	3.2	3.5	4.0
Eye length		mm	3.7±0.1(3.9^2^)	2.2±0.1(2.3^2^)	3.6	3.5
Maximum corneal facet diameter	D	µm	35.2±2.4	27.0±2.9	26	26
Corneal thickness		µm	110	50	60	80
Crystalline cone length		µm	60	40	40	60
Distal rhabdom diameter	d	µm	2.1	2.4	8	2
Rhabdom length	l	µm	370	240	300	350
Ommatidial length		µm	540	330	400	490
Focal length	f	µm	110±16	67±4	64±4	83±5
F-number	F		3.1	2.5	2.5	3.2
Theoretical acceptance angle	Δρ	°	1.0	2.1	7.2	1.4
Optical sensitivity	S	µm^2^sr	0.14	0.23	3.0	0.1

External measurements are from 4 V. crabro and 4 V. vulgaris.

1 data from Greiner 2006, except the intertegular width that was measured for this study, from 3 animals in the authors' collection.

2 data from Blackith 1958.

**Figure 3:**
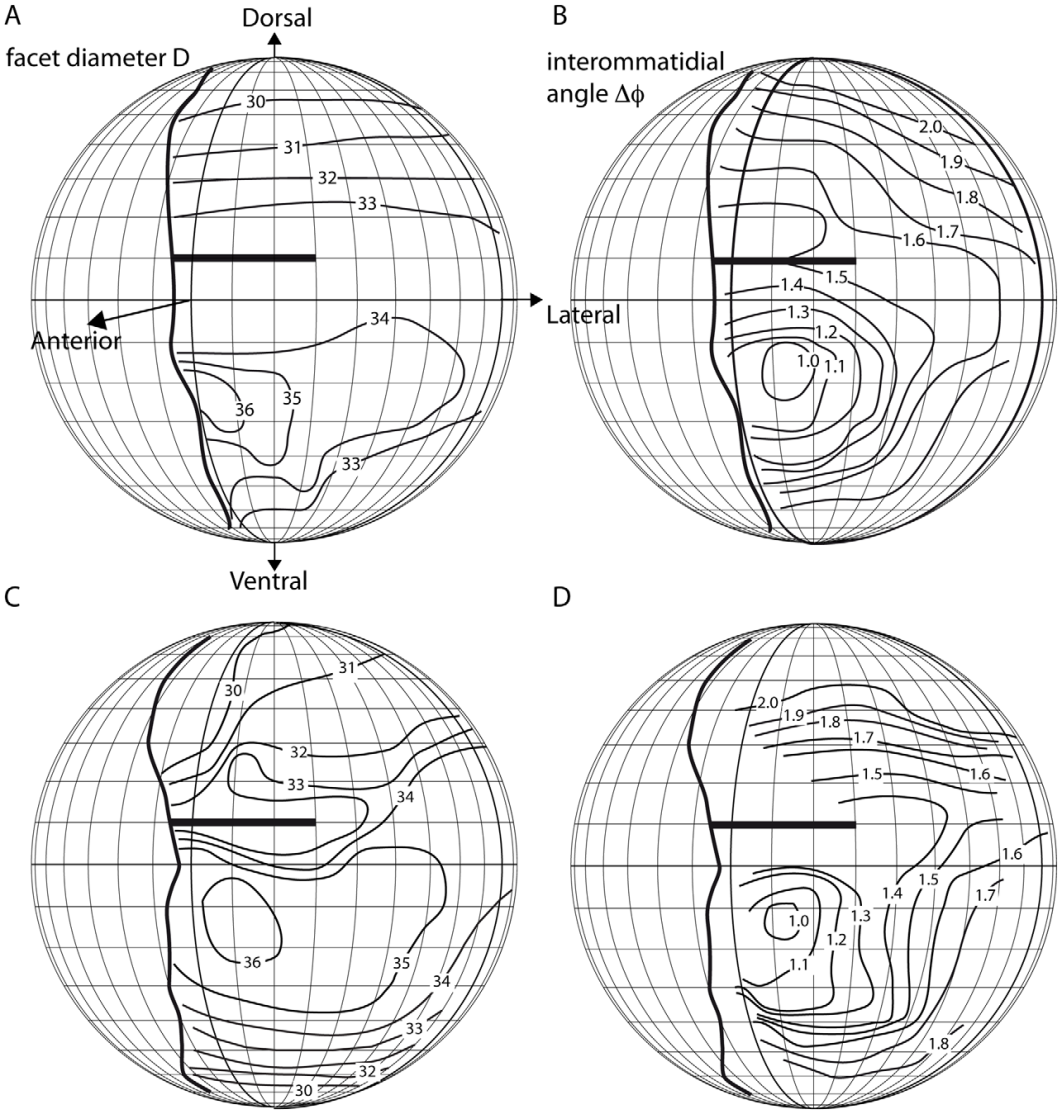
Contour maps of facet diameters *D* (A, C) and interommatidial angles Δϕ (B, D) in two *Vespa crabro* worker eyes. The thick vertical lines indicate the frontal visual field borders, anterior corresponds to latitude and longitude of 0°, dorsal to a latitude of +90°, ventral to a latitude of −90°, and lateral to a longitude of +90°. Facet diameters and interommatidial angles are indicated as isolines. In both animals, the ommatidia with the largest facet diameters (36 µm) also have the smallest interommatidial angles (1°) and look into a frontal and slightly ventral direction. The bold horizontal lines indicate the region in the visual field around +10° that is seen by ommatidia above and below the cuticular indentation into the hornet eye. Interommatidial angles in this region cannot be determined as accurately as in other eye regions.

*V. crabro* has an ommatidial length of 540 µm, with a remarkably thick cornea (110 µm), a 60 µm long crystalline cone and a 370 µm long rhabdom (Fig. 2C). The distal rhabdom diameter *d* is 2.1 µm (Fig. 2G), and the focal length *f* (measured using the hanging drop method and calculated with equation 1) is 110±16 µm (n = 8). The corneal facet diameter *D* is 35 to 36 µm in the fronto-ventral part of the eye (Fig. 2E, Fig. 3A, C) and decreases both laterally, dorsally and ventrally.

Corresponding values for *V. vulgaris* (Figure 1, Table 1) are an ommatidial length of 330 µm with a corneal thickness of 50 µm, a crystalline cone length of 40 µm and a rhabdom length of 240 µm. The distal rhabdom diameter is 2.4 µm, and hanging drop measurements of the focal length in *V. vulgaris* give a value of 67±4 µm (n = 8). The corneal facet diameter in the fronto-ventral part of the eye is 27 µm, but we were unable to map this parameter over the entire eye.

From the values of *f* and *D*, we calculated an F-number of 3.1 for the fronto-ventral part of the eye of *V. crabro* and 2.5 for *V. vulgaris*. The theoretical acceptance angle (Δρ) of the receptors in a fronto-ventral ommatidium are 1.0° for *V. crabro* and 2.1° for *V. vulgaris*. In *V. crabro*, the acceptance angle matches the interommatidial angles (Δϕ) in the frontal part of the eye (Fig. 3C, D). Interommatidial angles increase dorsally and ventrally.

Hornets, like all vespids, have an indentation on the frontal side of the eye (Fig. 1A, B). As a result of this, a small region of the visual field, at around 10° elevation, and from the contralateral border of the visual field to around 30° on the ipsilateral side (bold horizontal lines in Fig. 3) is seen twice, by ommatidia below and above the indentation. The consequences of this eye shape for vision has not been studied, and we found it difficult to determine interommatidial angles precisely in this part of the visual field that is seen twice by each eye. Within the ventral part of the eye, measurements were easier and more reliable, and we found that the region with the smallest interommatidial angles, and thus highest potential resolution, coincides with the area where ommatidia are largest and thus most sensitive, a combination indicating the presence of an acute zone.

The optical sensitivity (*S*) of ommatidia in this part of the eye, is 0.14 µm^2^sr for *V. crabro* and 0.23 µm^2^sr for *V. vulgaris*. Thus, as far as optics and the anatomy of the photoreceptors are concerned, *V. crabro* has a less sensitive eye than *V. vulgaris*. The difference is not large but comes as a surprise given the nocturnal flight activity of hornets.

### Ocelli

**Figure 4:**
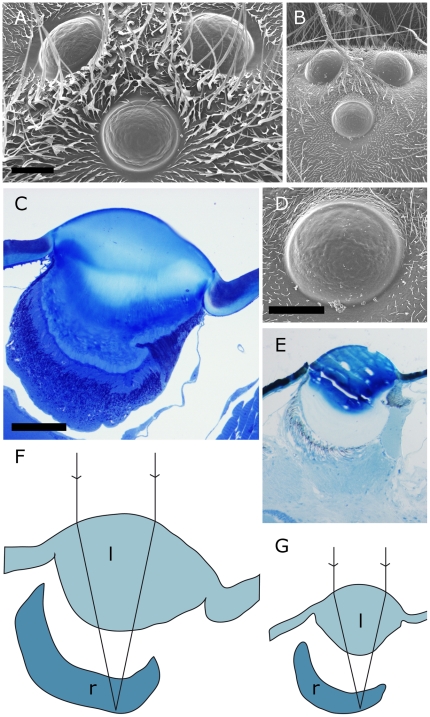
Ocelli of *Vespa crabro* (left side) and *Vespula vulgaris* (right side). Scales in each pair of figures are the same for both species. **A**, **B** Arrangement of all three ocelli on the head of the wasp, scale bar 200 µm. **C**, **E** Sagittal sections through the medial ocellus, scale bar 100 µm. **D** Close-up of the medial ocellus of *V. vulgaris*, scale bar 100 µm. **F**, **G** Focal length measured in hanging drop preparations superimposed on sketches of the medial ocelli indicating the lens (l) and retina (r) show that the focal plane lies within the retina. Same scale as **C** and **E**.

The three ocelli of *V. crabro* and *V. vulgaris* are situated on the top of the head, centred between the eyes (Figs 1, 4). In both species there were small differences in size between the lateral and the medial ocelli. The medial ocellus of *V. crabro* measures 359 µm in diameter and the lateral ocelli measure 336 µm in diameter. These are thus larger than the medial ocellus (207 µm) and the lateral ocelli (211 µm) of *V. vulgaris*, but relative to body size or head width, they are similar (Table 2).

**Table 2:** Ocellar size in four species of Vespidae.

	Symbol	Unit	*Vespa crabro*	*Vespula vulgaris*	*Apoica pallens^1^*	*Polistes occidentalis^1^*
Ocellar diameter						
Lateral	*D*	µm	336	211	414 / 486	161 / 218^1^
Medial	*D*	µm	359	207	416 / 427	216 / 273
Ocellar diameter/head width						
Lateral			0.060	0.066	0.117	0.056
Medial			0.064	0.065	0.120	0.070
Focal length						
Lateral	*f*	µm	292±74	149±10	331 / 450	185 / 190
Medial	*f*	µm	246±6	140±23	357 / 436	168 / 213
Back focal distance						
Lateral	*L*	µm	178±19	107±22	184 / 304	120 / 185
Medial	*L*	µm	145±19	104±19	162 / 190	100 / 170

Data from 5 *Vespa crabro* and 2 *Vespula vulgaris* are from this study. ^1^ Data from Warrant et al 2006. Ocelli of *Apoica pallens* and *Polistes occidentalis* are astigmatic and the values for the long and short axes are given.

The ocellar focal lengths determined for *V. crabro* (n = 5) were 292±74 µm for the lateral ocelli and 246±6 µm for the medial ocelli. For *V. vulgaris* (n = 2) the ocellar focal lengths were 149±10 µm for the lateral ocelli and 140±23 µm for the medial ocellus. The optical back focal distance was 178±19 µm for the lateral ocelli and 145±19 µm for the medial ocellus of *V. crabro*. Corresponding values for *V. vulgaris* were 107±22 µm and 104±19 µm, respectively. Superimposing these distances onto longitudinal sections of the ocelli shows that images formed by the ocellar lens are focussed within the retina (Fig. 4G, H).

## Discussion

### Hornet and wasp flight activity

On moonless nights and nights with a quarter moon, hornets are active until late after sunset, as long as pale objects have a luminance of between 0.01 and 0.1 cd/m^2^. In detail, they fly a little later than the end of civil twilight (which is when the sun is 6° below the horizon) but not as late as the end of nautical twilight (which is when the sun is 12° below the horizon, see Fig. 1A, B). Our observations of hornet flight activity confirm the results reported by Blackith [1]. He measured light levels in lumens/squarefoot, which are difficult to compare with our luminance measurements. Like Blackith [1], we find that hornets should also be able to fly on nights with a full or three quarter moon. Common wasps, as Blackith [1] reports, only fly at light intensities that are about one hundred times brighter, and are thus restricted to the brighter part of civil twilight. Blackith's [1] data show that the activity of the smaller wasps is restricted mainly by light intensity and not by low temperature because he measured air temperature at the same time as he measured light intensity and found that it differed both between days and between last flights in the evening and first flights in the morning, while light levels remained relatively constant. Still, we cannot completely exclude some influence of temperature or other factors such as food supply on the activity that we observed.

### Hornet eyes have no adaptations to vision in dim light

The obvious difference in flight activity between hornets and wasps is not reflected in the adaptations of the eyes. Hornets have slightly less sensitive eyes than wasps, but the optical sensitivity of the eyes of both species is similar to that of another exclusively diurnal wasp, *Polistes occidentalis* reported by Greiner [4] (see Table 1 for the comparison). The eyes of *V. crabro* have larger facet lenses and a longer focal length, resulting in F-numbers that are similar to those of both diurnal species. Even the eyes of *Apoica pallens*, the only species that restricts flight activity to the night, have a similar F-number. That *A. pallens* has more sensitive eyes results only from one specific adaptation: they have 8 µm wide rhabdoms resulting in acceptance angles in the fronto-ventral ommatidia of 7°. For comparison, *V. vulgaris* has acceptance angles of 2°, in the same region of the eye, and *V. crabro* has even smaller acceptance angles, down to 1°.

### High resolution and the potential to see well at night – the advantage of being large

An optical sensitivity similar to that found in *V. crabro* (0.14 µm^2^sr) is found in diurnal bees (*Apis mellifera*, 0.1), in humans (0.1; for review see [24]), and also in two large bee species that are normally diurnal but are able to forage on moonlit nights: *A. dorsata*, the Asian giant honeybee [8], and *X. tenuiscapa*, a large Indian carpenter bee [7]. Both species also have narrow rhabdoms and small acceptance angles, similar to *V. crabro*, and while interommatidial angles are not known for *A. dorsata*, those of *X. tenuiscapa* are as small as those of *V. crabro* [7].

All mentioned species have their relatively large body size and accordingly large eye size in common. Larger eyes allow for more ommatidia and larger facet lenses, and thus for higher resolution and higher sensitivity. In species that are active in dim light, we expect higher sensitivity to be favoured at the cost of high resolution. Thus, we might expect these species to have relatively few ommatidia with large facets.

When studying bees that forage in the extremely dim light of a rain forest at dusk (*M. genalis*; 0.0001 cd/m^2^ [5]) or a moonless night (*X. tranquebarica*, 0.00001 cd/m^2^ [12]), we were surprised to find that their eyes were only about 30 times more sensitive than those of diurnal bees and showed very small interommatidial angles. This seemed paradoxical, as the large acceptance angles of the ommatidia result in oversampling. This compromises the high resolution that is theoretically possible with the small interommatidial angles. Additional studies indicated that, in *M. genalis*, spatial summation of signals is likely to increase the sensitivity neuronally, at the cost of spatial resolution [3], [13]. This solution – high spatial resolution and later neuronal pooling of signals – does not seem optimal for an obligately nocturnal animal. However, if we consider that obligately nocturnal behaviour of bees has very likely evolved from facultative crepuscular activity, we see a different picture.

Only animals with large eyes – associated with large body size – could take advantage of spatial pooling without loosing too much resolution. This gave large bees access to nectar and pollen sources not available to small bees during the brighter hours of day. Spatial pooling is likely to be the first adaptation to dim light vision that evolved in bees such as *X. tenuiscapa* and *A. dorsata*, and as pooling probably only occurs at night, this strategy allowed the insects to use the full spatial resolution of their eyes during the day. Only bees that turned to a completely nocturnal lifestyle, like *X. tranquebarica* and *M. genalis*, evolved the large rhabdom diameters that destroyed the high resolution of the ommatidial array, allowing these bees to fly at even dimmer light intensities. Our present study on hornets confirms this hypothesis for wasps and leads us to speculate that the obligate nocturnal behaviour and wide rhabdoms of *A. pallens* may have evolved via a stage of facultative twilight activity, as in *V. crabro*. Similar to the facultatively crepuscular bees, the single ommatidia of *V. crabro* have a rather low optical sensitivity, and we must assume that they use spatial (and/or temporal) summation to be able to fly in dim light, compromising spatial and temporal resolution during the dark hours. To what exact degree pooling is used, we can only speculate at this point.

The first behavioural evidence for temporal pooling comes from a study showing that tethered hornets, like the nocturnal bee *M. genalis* [25], fly more slowly in dim light than in bright light [2]. Nonetheless, further investigations on hunting behaviour, flight control and information processing in the optic pathway of hornets are needed. We are now planning to measure spatio-temporal resolution of hornets and wasps flying at different light levels. The only species for which a relationship between spatial resolution and light intensity has been measured and given clear evidence of pooling, is the honeybee *Apis mellifera* [14].

In a study on the crepuscular butterfly *Caligo memnon*, Frederiksen and Warrant [26] suggest that the evolution of crepuscular and nocturnal vision with apposition eyes of wide rhabdoms started with large body size – and thus eye size – and continued with the evolution of wide rhabdoms. We can also see that facultatively nocturnal bees and wasps have not taken this second step yet. Our data strongly suggest that – at least in hymenoptera – neural summation must be present before the evolution of wide rhabdoms, and we may have to wait for more intermediate forms on this evolutionary path to be discovered.

Besides eye size, another advantage of a large body size is the ability to maintain a higher temperature. We also plan to study body temperature of flying wasps and hornets to better understand the effect of body size on the ability to keep a high enough temperature for efficient flight.

### Wasp ocelli – visual organs with potential for spatial resolution?

Earlier studies show a strong connection between ocellar size and dim light activity in hymenopterans [5], [11], [27]. Species active in dim light conditions have larger ocelli than those active in bright light. The ocelli of *V. crabro* are big, but not as big as those of *A. pallens.* In fact, relative to body and head size, the ocellar size is much more similar to that of the diurnal wasps *V. vulgaris* and *P. occidentalis*. It is unlikely that the ocelli of *V. crabro* are specifically adapted for dim light conditions. The large ocellar size, like the large eye size, is a consequence of the large body size, as is also the case in the large carpenter bee *X. tenuiscapa*.

However, another result deserves attention. Like the other studied wasps *A. pallens* and *P. occidentalis* [11], both *V. crabro* and *V. vulgaris* have ocelli with a rather short focal length, short enough for the plane of focus to lie within the retina (Fig. 4). Theoretically this allows them to produce an image with some retinal resolution, unlike the ocelli of bees and many other insects that are severely underfocused and thought to measure general light intensity [28]. Closer investigations of the anatomy and physiology of the ocellar photoreceptors, and subsequent interneurons, are required to understand whether this really is the case. So far, the only insects, which are known to have and use the resolving power of their ocelli to form a coarse image are dragonflies [29].

### Conclusions

We confirm here the finding of Blackith [1] that European hornets, *Vespa crabro*, fly in dim twilight down to intensities of 0.001 cd/m^2^ and can thus even fly in bright moonlight. They can do this even though the optics and anatomy of their eyes and ocelli show none of the optical or anatomical adaptations typically found in truly nocturnal hymenoptera. In contrast, their eyes and ocelli generally seem very similar to those of the diurnal wasps *V. vulgaris* and *P. occidentalis*. This is similar to findings in facultatively nocturnal bees. We propose that neuronal summation is the only possible way for hornets to achieve the necessary sensitivity that allows them to fly in dim light, and suggest that summation generally precedes anatomical adaptations such as widened rhabdoms, when hymenopteran insects change from a diurnal to a nocturnal lifestyle.
